# Does ultrasound imaging of the spastic muscle have an additive effect on clinical examination tools in patients with cerebral palsy?: A pilot study

**DOI:** 10.14744/10.14744/nci.2020.78045

**Published:** 2022-03-11

**Authors:** Kardelen Gencer Atalay, Evrim Karadag Saygi, Firat Akbas, Ozge Kenis Coskun, Ahmet Hamdi Akgulle, Ilker Yagci

**Affiliations:** 1 Department of Physical Medicine and Rehabilitation, Marmara University School of Medicine, Istanbul, Turkey; 2 Department of Orthopedics and Traumatology, Marmara University School of Medicine, Istanbul, Turkey

**Keywords:** Gastrocnemius, spastic cerebral palsy, ultrasound imaging

## Abstract

**Objective:**

The Modified Ashworth Scale, the Modified Tardieu Scale, and measuring the passive range of motion is commonly preferred examination tools for spasticity in cerebral palsy (CP). Ultrasonography has become increasingly used to provide relevant insight into spastic muscle morphology and structure recently. It was aimed to reveal associations between the clinical and ultrasonographic parameters of gastrocnemius medialis (GM) and lateralis muscles in this population.

**Methods:**

Thirty-four children with spastic CP aged between 4 and 12 years who did not have botulinum neurotoxin A intervention within 6 months or had no previous history of any orthopedic or neurological surgery were included. The spasticity of GM and lateralis was evaluated firstly by the Modified Ashworth Scale, Modified Tardieu Scale, and ankle passive range of motion. Then, the cross-sectional area (CSA), muscle thickness (MT), qualitative and quantitative echo intensity (EI) values of both muscles were measured from their ultrasonographic images.

**Results:**

The CSA of GM, and qualitative EI of both muscles were found to be mild-to-moderately correlated to all clinical examination tools (p<0.01), whereas the CSA of gastrocnemius lateralis was mildly related to Modified Ashworth Scale (p=0.009). The MT and quantitative EI of both muscles were not associated with any of the clinical tools (p>0.05).

**Conclusion:**

Ultrasonographic measurements of GM and lateralis partially reflect ankle spasticity in children with CP. Ultrasonography can be used as an alternative tool in this patient population where the clinical evaluation can not perform ideally.

Cerebral palsy (CP) is a group of motor disorders caused by an abnormality or non-progressive injury to the developing brain. Spasticity, one of the most common impairments of CP, is characterized by a velocity-dependent increase in the tonic stretch of muscles [1]. This change in tonus, along with other impairments such as disuse and immobilization, results in morphological and structural alterations and related joints’ passive range of motion (PROM) restrictions, or contractures. The Modified Ashworth Scale (MAS) and the Modified Tardieu Scale (MTS) are the most commonly used clinical tools for spasticity evaluation [1, 2]. Despite their frequency of use, these assessments are criticized for being oversimplified and insufficient. The subjective nature of them leads to limited intra- and inter-rater reliability [3–6]. Moreover, they can not always be performed ideally, especially in the pediatric patient population. Thus, imaging modalities are rising to prominence with more relevant insight into the condition by quantifying morphological and structural alterations of the muscle. Magnetic resonance imaging techniques and three-dimensional (3D) ultrasonography systems can capture these alterations objectively with satisfactory reliability results. 2D ultrasonography, due to its easy accessibility and relatively low-cost, has been widely preferred in the studies [7, 8].

The gastrocnemius medialis (GM) in children with spastic CP has been reported consistent evidence of reduced muscle size indicated by volume, cross-sectional area (CSA), muscle thickness (MT), or belly length [1, 9–12]. In addition to these well-documented gross morphological changes, a few studies have also examined GM muscle composition indicated by echo intensity (EI) that reflects the amount of intramuscular fat and collagen, possibly contributing to weakness and stiffness. Both qualitative and quantitative EI values have been found to be higher in spastic CP compared to typically developing children [7, 10, 13]. Recently, the use of botulinum neurotoxin A (BoNT-A), which is a common treatment in spastic CP, is blamed for inducing chemodenervation-induced atrophy and for causing more apparent alterations in muscle morphology and composition. In a study that a 3D ultrasonography system was used to obtain the GM volume and echo-intensity, significant alterations were found in the spastic CP cohort compared to typically developing children. These alterations were shown to be highly associated with BoNT-A history and Gross Motor Function Classification System (GMFCS) level [14]. This study aims to investigate the relationship between the clinical and ultrasonographic parameters that are achieved from 2D ultrasonography in children with spastic CP. The hypothesis is that CSA, MT, and EI of GM and gastrocnemius lateralis (GL) are associated with ankle PROM and spasticity, and concurrently correlated to patients’ number of BoNT-A interventions and GMFCS levels.

## Materials and Methods

This study was conducted between October 2016 and January 2019 at Marmara University Physical Medicine and Rehabilitation Department, Istanbul, Turkey. Children with spastic CP, whose diagnosis was confirmed by cranial magnetic resonance imaging and clinical examination were included. The study was approved by the local ethics committee (Marmara University School of Medicine, 09.2011.0047), and informed consent was obtained from the parents of all participants. The inclusion criteria were determined as follows: (1) Age between 4 years and 12 years; (2) naive to BoNT-A interventions or at least 6 months after the last BoNT-A intervention; (3) no previous orthopedic or neurological surgery.

**Highlight key points**

• Subjective clinical assessment tools for spasticity can not always be performed ideally in the pediatric patient population.
• Ultrasound measurements of gastrocnemius medialis and gastrocnemius lateralis were found to be correlated with ankle passive range of motion and stiffness.
• The Gross Motor Function Classification System level of the patients was found to be significantly related to the ultrasound measurements.

Patients’ age, gender, weight, height, type of CP (hemiplegia, diplegia, and quadriplegia), GMFCS level, and BoNT-A history were recorded. Ankle PROM of the involved leg (affected leg in hemiplegia, both legs in diplegia, and quadriplegia) was measured by a 4-year trained physiatry resident (FA) using a handheld goniometer in the supine position while the knees were fully extended. Both dorsiflexion and plantarflexion angles were measured as positive, considering the neutral position of the ankle as 0°. It was measured as negative, in case of the PROM angle was more limited than the neutral position. The gastrocnemius muscle spasticity was evaluated by the same examiner (FA) using MAS and MTS also in the supine position while the knees were fully extended. The MAS, 0–4 grading scale based on assessing the resistance to passive stretch of a relaxed joint, (0, no increase in muscle tone; 1, slight increase in muscle tone at the end of the range of motion; 1+, slight increase in muscle tone through less than half of the range of motion; 2, more marked increase in muscle tone through most of the range of motion; 3, considerable increase in muscle tone; 4, joint is rigid), was considered 0–5 (0=0; 1=1; 1+=2; 2=3; 3=4; 4=5) for statistical purpose [5, 15]. The MTS was used to measure spasticity angles (R1, R2) at which the muscle reaction occurred at two different velocities: R2 was defined as the full range of motion during a slow-velocity, where R1 as the threshold angle of catch-and-release or clonus during a fast-velocity. The difference between these angles (R2-R1) was calculated to reflect the dynamic component of spasticity [3, 16].

**Figure 1.**
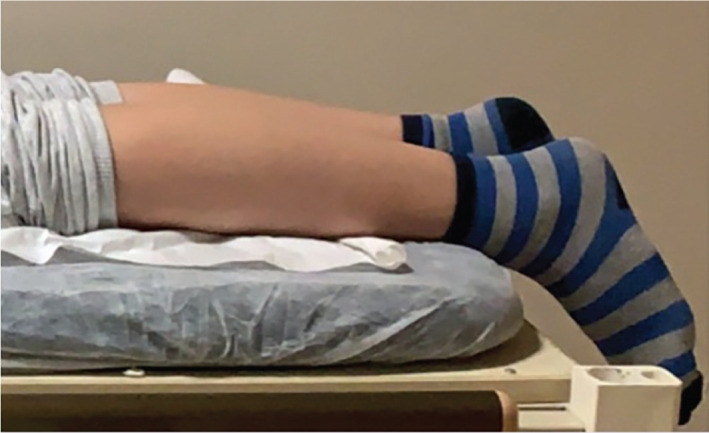
The position of the patient on the examination table while performing ultrasound measurements.

**Figure 2.**
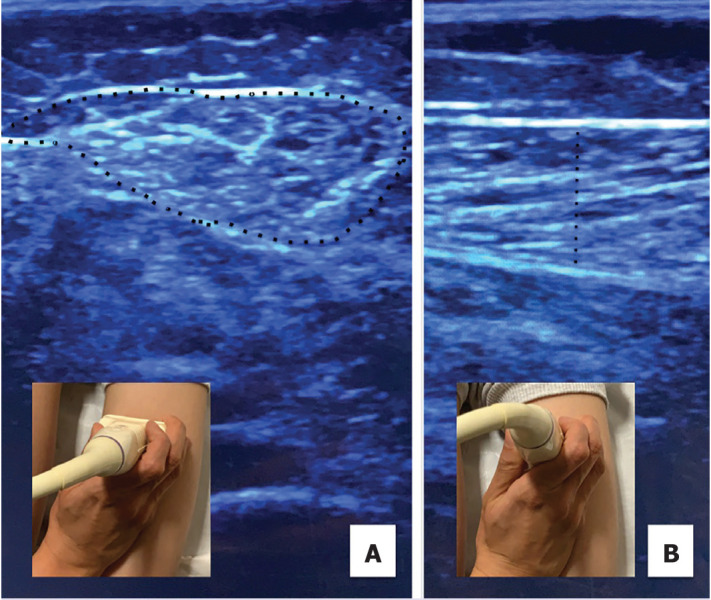
**(A)** Cross-sectional area of gastrocnemius medialis (This image represents grade 2 echo intensity according to I-IV Heckmatt Scale), **(B)** Muscle thickness of gastrocnemius medialis.

Ultrasound measurements were performed by a physiatry specialist with 5 years of experience in musculoskeletal ultrasonography (KGA) from the most prominent bulge of the GM and GL of the involved leg while the ankle in a resting position with the knee fully extended. Each patient lay prone on the examination table with the feet hanging over the edge. The ankle angle was fixed at an approximate plantarflexion angle of 20° [5, 7] (Fig. 1). The CSA (mm^2^) in the transverse view and MT (mm) in the longitudinal view were measured using a 6–18 MHz linear array probe (Logiq P5, General Electric, Boston, MA, USA) (Fig. 2a, b). The probe was held with a light touch in order not to cause any difference in the muscle size. The CSA of both muscles was normalized to body mass (mm^2^/kg) to investigate associations [17]. Since EI is known to be influenced by the ultrasound acquisition settings, care was taken to ensure frequency (10 MHz), depth (5.5 cm), focus (1.8–2.8 cm), gain (38%), dynamic range (44 dB), and unaltered time-gain compensation kept constant [14]. The qualitative EI was visually graded using I-IV Heckmatt Scale in the transverse view by two different examiners (KGA, FA): Grade I is normal, grade II represents an increase in EI while bone echo is still distinct, grade III indicates a marked increase in muscle EI with a reduced bone echo and grade IV indicates a very high muscle EI and a complete loss of bone echo [13]. The quantitative EI (expressed in a grey-scale of 256 values) was obtained from the first examiner’s ultrasonographic image by excluding data outside the muscle region of interest and then exporting in jpeg format into MATLAB (R2015b, The MathWorks, Natick, MA, USA) for 2-D image histogram-based analysis [10].

### Statistical Analysis

Statistical analysis was performed with a statistical software package (SPSS Inc. Released 2009. PASW Statistics for Windows, Version 18.0. Chicago, USA). The normal distribution of quantitative values was assessed using a histogram, a Q-Q plot, and the Shapiro-Wilk test. Spearman’s rho correlation analysis was performed to examine the relationship between ultrasonographic findings, clinical examination tools, BoNT-A history, and GMFCS level. The strength of the correlation was described as weak when the absolute value of rho between 0.20 and 0.39, whereas moderate between 0.40 and 0.59 and strong between 0.60 and 0.79. The intraclass correlation coefficient (ICC) estimates and their 95% confidence intervals for inter-rater reliability analysis of the I-IV Heckmatt Scale for GM and GL were calculated based on single measurement, consistency, 2-way random-effects model. A statistical significance level of p<0.05 was accepted.

## Results

A total of 50 children with spastic CP aged between 4 and 12 years were assessed for eligibility. Of these, 16 children were excluded because of previous orthopedic surgery (n=9), BoNT-A intervention within the last 6 months (n=6), and refused to participate (n=1). The demographic and clinical characteristics of 34 patients included in the study are presented in Table 1. The ankle PROM, gastrocnemius spasticity, and ultrasound measurements of their 61 involved legs (affected leg in hemiplegia, both legs in diplegia and quadriplegia) are shown in Table 2.

**Table 1.** Demographic and clinical characteristics of the study population (n=34)

Characteristic	Patients
Age (month)	75.59±29.3
Gender (F/M)	47.1/52.9
Weight (kg)	19.13±6.27
Height (m)	1.07±0.13
Type of cerebral palsy	
Hemiplegia	20.6
Diplegia	41.2
Quadriplegia	38.2
GMFCS level
I	17.6
II	20.6
III	17.6
IV	20.6
V	23.5
BoNT-A intervention (>6 mo)	
0	14.7
1	35.3
2	26.5
3	14.7
4	5.9
5	2.9

Data=(%), Mean±SD; GMFCS: Gross motor function classification system, BoNT-A: Botulinum neurotoxin A.

**Table 2.** Clinical features and ultrasound measurements of patients’ involved legs (n=61)

Clinical examination tools	Median (95% CI)	Min–Max
Ankle dorsiflexion PROM (deg)	-25 (-29.9–18.75)	-60–15
Ankle plantarflexion PROM (deg)	60 (51.42–60.06)	-25–80
MAS (0-5)	2 (2.2–2.68)	0–5
MTS (deg)		
R1	-20 (-23.35–12.23)	-70–60
R2-R1	25 (14.36–30.33)	-105–100
Ultrasound measurements	GM	GL
	Median (95% CI)	Median (95% CI)
CSA (mm^2^)	262.54 (248.9–14.73)	177.11 (169.25–221.47)
CSA-norm (mm^2^/kg)	14.55 (13.5–15.9)	8.88 (9.07–11.37)
MT (mm) echo intensity	7.3 (7.1–8.38)	6.2 (6.11–7.37)
Heckmatt Scale ((I-IV)	2 (2.11–2.52)	3 (2.55–2.93)
Histogram-based analysis (0–255)	139.95 (130.05–150.65)	143.98 (135.35–159.16)

PROM: Passive range of motion; MAS: Modified ashworth scale; MTS: Modified Tardieu scale; CSA: Cross-sectional area; CSA-norm: Cross-sectional area normalized to body mass; MT: Muscle thickness; GM: Gastrocnemius medialis; GL: Gastrocnemius lateralis; CI: Confidence interval; Min: Minimum; Max: Maximum.

### Associations between Ultrasound Measurements and Clinical Examination Tools

Thirty-four patients’ 61 involved leg outcomes were included to investigate associations between ultrasound measurements and clinical examination tools. Mild to moderate significant correlations were found for GM, whereas weaker associations for GL. Normalized CSA of GM was positively correlated with the ankle dorsiflexion PROM. Normalized CSA of both muscles was negatively correlated with MAS. The qualitative EI grades of both muscles were negatively correlated with the ankle dorsiflexion PROM and the difference between MTS spasticity angles (R2-R1), while positively correlated with MAS and MTS spasticity angle at a fast-velocity (R1). On the other hand, the quantitative EI value of GM was only positively correlated with the MTS spasticity angle at a fast-velocity (R1) and negatively correlated with the difference between MTS spasticity angles (R2-R1) (Table 3).

**Table 3.** The significant associations between ultrasound measurements and clinical examination tools

	Gastrocnemius medialis				Gastrocnemius lateralis			
	CSA-norm (mm^2^/kg)	MT (mm)	Echo intensity Heckmatt Scale (I-IV)	Echo intensity Histogram-based analysis (0–255)	CSA-norm (mm^2^/kg)	MT (mm)	Echo intensity Heckmatt Scale (I-IV)	Echo intensity analysis (0–255)
Ankle dorsiflexion PROM (deg)	0.341, 0.007**		-0.405, 0.002**				-0.285, 0.038*	
Ankle plantarflexion PROM (deg)								
MAS (0-5)	-0.434, p<0.001		0.489, p<0.001		-0.333, 0.009**		0.278, 0.044*	
MTS (deg)								
R1			0.498, p<0.001	0.345, 0.009**			0.395, 0.003**	
R2-R1	0.255, 0.047*		-0.597, p<0.001	-0.322, 0.014*			-0.475, p<0.001	

Data: rho, P-value. PROM: Passive range of motion; MAS: Modified Ashworth Scale; MTS: Modified Tardieu Scale; CSA-norm: Cross-sectional area normalized to body mass; MT: Muscle thickness; *: P<0.05; **: P<0.01.

### Association between Ultrasound Measurements and BoNT-A History, and GMFCS Level

Only one leg of the patients, in the case of both legs involvement, the right one was chosen, was included to investigate associations between ultrasound measurements and BoNT-A history and GMFCS level. The number of BoNT-A interventions was not found to be associated with any of the ultrasound measurements of GM, but only moderately correlated with the qualitative EI grade of GL. The GMFCS level had strong negative correlations with normalized CSA and thickness of GM, a moderate negative correlation with normalized CSA of GL, and moderate positive correlations with the qualitative EI grades of both muscles (Table 4).

**Table 4.** The significant associations between ultrasound measurements and BoNT-A history, and GMFCS level

	Gastrocnemius medialis				Gastrocnemius lateralis			
	CSA-norm (mm^2^/kg)	MT (mm)	Echo intensity Heckmatt Scale (I-IV)	Echo intensity Histogram-based analysis (0–255)	CSA-norm (mm^2^/kg)	MT (mm)	Echo intensity Heckmatt Scale (I-IV)	Echo intensity Histogram-based analysis (0–255)
Number of BoNT-A intervention (0–5)							0.582, p<0.001	
GMFCS level (I-V)	-0.711, p<0.001	-0.719, p<0.001	0.432, 0.014*		-0.495, 0.003**		0.548, 0.001**	

Data: rho, P-value. GMFCS: Gross Motor Function Classification System; CSA-norm: Cross-sectional area normalized to body mass; MT: Muscle thickness; BoNT-A: Botulinum neurotoxin A; *: P<0.05, **: P<0.01.

### Inter-observer Reliability of Qualitative EI Grading (I-IV Heckmatt Scale)

Inter-observer reliability of the I-IV Heckmatt Scale was shown to be good for both GM and GL; ICC (95%CI) was found to be 0.725 (0.527, 0.841) and 0.706 (0.491, 0.830), respectively. In addition, the I-IV Heckmatt Scale of both muscles was found to be moderately correlated to quantitative histogram-based EI analyses (rho=0.582, 0.560, p<0.001 for GM and GL, respectively).

## Discussion

This study showed varying degrees of correlations between ultrasound measurements of GM and GL muscles, and ankle PROM and spasticity. The normalized CSA of GM and EI of both GM and GL were found to be associated with ankle dorsiflexion PROM, MAS, and MTS, whereas the normalized CSA of GL was only related to MAS. These results were consistent with the outcomes of studies in adult patients with stroke [15, 18, 19]. However, muscle size and spasticity association could not be shown in the previous studies that included children with spastic CP [11]. Muscle size, especially in children with CP, was proven to differ prominently between subjects depending on body size, therefore, preferring normalized values were suggested to reduce the heterogeneity when investigating muscle architecture [17]. The relationship between muscle size and spasticity could have been overshadowed because normalized values were not used in the previous studies. Indeed, in this study, spasticity was found to be associated with the normalized CSA of both muscles. Thus, it was not related to the MT that could not be normalized due to the lack of an identified equation. Muscle EI, contrary to muscle size, was not recommended to be corrected depending on age, height, or weight, because of their slight relationship [20]. Consequently, in this study, along with previous ones that included children with CP, EI was found to be significantly correlated with ankle PROM and stiffness [13].

The number of BoNT-A interventions applied in the past was found to be associated with only qualitative EI of GL, but none of the ultrasound measurements of GM. This result was inconsistent with the outcome of the study by Schless et al. [14] because of two potential reasons. The first one could be the difference in the BoNT-A intervention numbers of the studies. Schless et al. included patients with a minimum of three recurrent BoNT-A interventions into the BoNT-A group, whereas 72.5% of patients included in this study undergone one or two BoNT-A interventions. Fewer interventions might only have affected GL, a smaller muscle in size, but not the GM. The second one could be the extensive detection ability of 3D ultrasonography on determining structural changes when compared to a 2D ultrasonography image. Finally, although the effect of BoNT-A history on EI could not have been shown directly in this study, the inverse relationship found between EI and the spasticity angle difference of MTS (R2-R1) could support the evidence of BoNT-A response reduction in muscles with increased EI [21].

The GMFCS level was found to be significantly related to muscle size and EI of both GM and GL. These results confirmed the study by Schless et al. yet were in contrast to the study by Battisti et al., which found the level of GMFCS was only associated with EI of soleus, not GM and GL [13, 14]. This discrepancy might have been caused by the exclusion of GMFCS level V patients in the Battisti et al. [13] study. Since soleus was not examined in this study, whether it was affected more than GM or GL depending on GMFCS level was remained ambiguous.

In this study, both the qualitative and quantitative EI were applied to the same ultrasonography images, and it was shown that the qualitative EI grades with sufficient inter-rater reliability reflected clinical outcomes better than the quantitative EI values. This finding was considerably unexpected by reason of the qualitative evaluation of EI that compared intramuscular tissue to the bone was always considered to be a simple, naked-eye assessment while the quantitative evaluation was offering a more objective further analysis [22]. Accordingly, a similar result was found in a study that included adult patients with amyotrophic lateral sclerosis, and it was attributed to the difference between categorical and scale assessments [23]. Both studies supported the evidence of the qualitative EI was more specific while the quantitative EI more sensitive [13].

Limitations of this study could be listed as relatively small sample size and lack of a control group. However, the sample size was comparable to the previously reported research designs, and a control group was not necessarily due to constant confirmation of muscle size and EI evidence in this patient group. In addition to these, the relatively low number of patients’ BoNT-A interventions might have masked the association between structural changes and BoNT-A history.

## Conclusion

Ultrasonographic measurements of both GM and GL in children with spastic CP were found to be associated with spasticity of the ankle and GMFCS level. Even though 3D ultrasonography systems can capture the alteration more accurately, 2D ultrasonography can be preferred as an alternative tool in children with spastic CP, where the clinical evaluation can not perform ideally. However, the lack of association between these measurements and BoNT-A intervention raises doubts about the clinical usefulness without examination.
